# Primary Gastrointestinal Follicular Lymphoma Presenting With Bowel Stenosis

**DOI:** 10.7759/cureus.21278

**Published:** 2022-01-15

**Authors:** Hiroto Suzuki, Yasuhiko Hamada, Kyosuke Tanaka, Noriyuki Horiki, Hayato Nakagawa

**Affiliations:** 1 Department of Gastroenterology and Hepatology, Mie University Hospital, Tsu, JPN

**Keywords:** stenosis, small bowel, follicular lymphoma, endoscopy, double-balloon enteroscopy

## Abstract

Primary follicular lymphomas of the small bowel generally present with small whitish nodules and masses or polyp-like lesions; cases with other morphologies are extremely rare. We experienced a case of primary follicular lymphoma that presented with small bowel stenosis. The lesion needed to be differentiated from other causes, such as bowel tuberculosis, non-steroidal anti-inflammatory drug-related ulcers, Crohn’s disease, small bowel ischemia, trauma, and idiopathic bowel stenosis, but endoscopic biopsies did not result in a definite diagnosis. Therefore, the lesion was surgically resected and, consequently, a diagnosis of follicular lymphoma of the small bowel was finally made. We report the characteristics and macroscopic findings of follicular lymphoma of the small bowel along with a review of relevant literature.

## Introduction

The gastrointestinal tract is the most common site of extranodal non-Hodgkin’s lymphoma (NHL), and primary gastrointestinal NHL accounts for 30-40% of all extranodal NHL cases [1]. The most common sub-types of primary gastrointestinal NHL are mucosal-associated lymphoid tissue lymphoma or diffuse large B-cell lymphoma, while follicular lymphoma (FL) is a relatively rare disease, accounting for approximately 1-3% of gastrointestinal NHL cases [2-4]. Endoscopic findings of gastrointestinal FL primarily include scattered small whitish nodules and polyp-like lesions. However, other morphologies are exceedingly rare [4]. Recently, the advances in endoscopic techniques and devices, such as double-balloon enteroscopy (DBE) and video capsule endoscopy (VCE), have allowed gastroenterologists to detect atypical lesions in patients with gastrointestinal FL [5].

Here, we report an unusual case of gastrointestinal FL that presented with small bowel stenosis, with the characteristics and macroscopic findings of small bowel FL, along with a review of the relevant literature.

## Case presentation

A 73-year-old man presented with a four-week history of emesis, with no history of abdominal surgery, nonsteroidal anti-inflammatory drug (NSAID) use, Crohn’s disease, and trauma, and no relevant family history of other medical conditions. Physical examinations revealed abdominal distension, while laboratory tests revealed a slight decrease in the level of hemoglobin and mean corpuscular volume and a slight increase in the level of the soluble interleukin-2 receptor (Table 1). 

**Table 1:** Laboratory results.

Laboratory item	Result	Normal range
White blood cell count	3410×10^3^/mL	3300–8600×10^3^/mL
・Neutrophils	41.6%	37.0–72.0%
・Lymphocytes	45.2%	20.0–50.0%
・Monocytes	8.2%	4.1–10.6%
・Eosinophils	3.8%	0.6–8.3%
・Basophils	1.2%	0.0–1.3%
Hemoglobin	12.4 g/dL	13.7–16.8 g/dL
Mean corpuscular volume	82.3 fL	83.6–98.2 fL
Platelet	280×10^4^ g/dL	158–348×10^4^ g/dL
Blood urea nitrogen	7.8 mg/dL	8.0–20 mg/dL
Creatinine	0.75 mg/dL	0.65–1.07 mg/dL
Total bilirubin	0.7 mg/dL	0.4–1.5 mg/dL
Alkaline phosphatase	166 U/L	106–322 U/L
Lactate dehydrogenase	143 U/L	124–222 U/L
Aspartate transaminase	16 U/L	13–30 U/L
Alanine aminotransferase	12 U/L	10–42 U/L
C-reactive protein	0.06 mg/dL	< 0.14 mg/dL
Soluble interleukin-2 receptor	549 U/mL	122–496 U/mL

**Figure 1:**
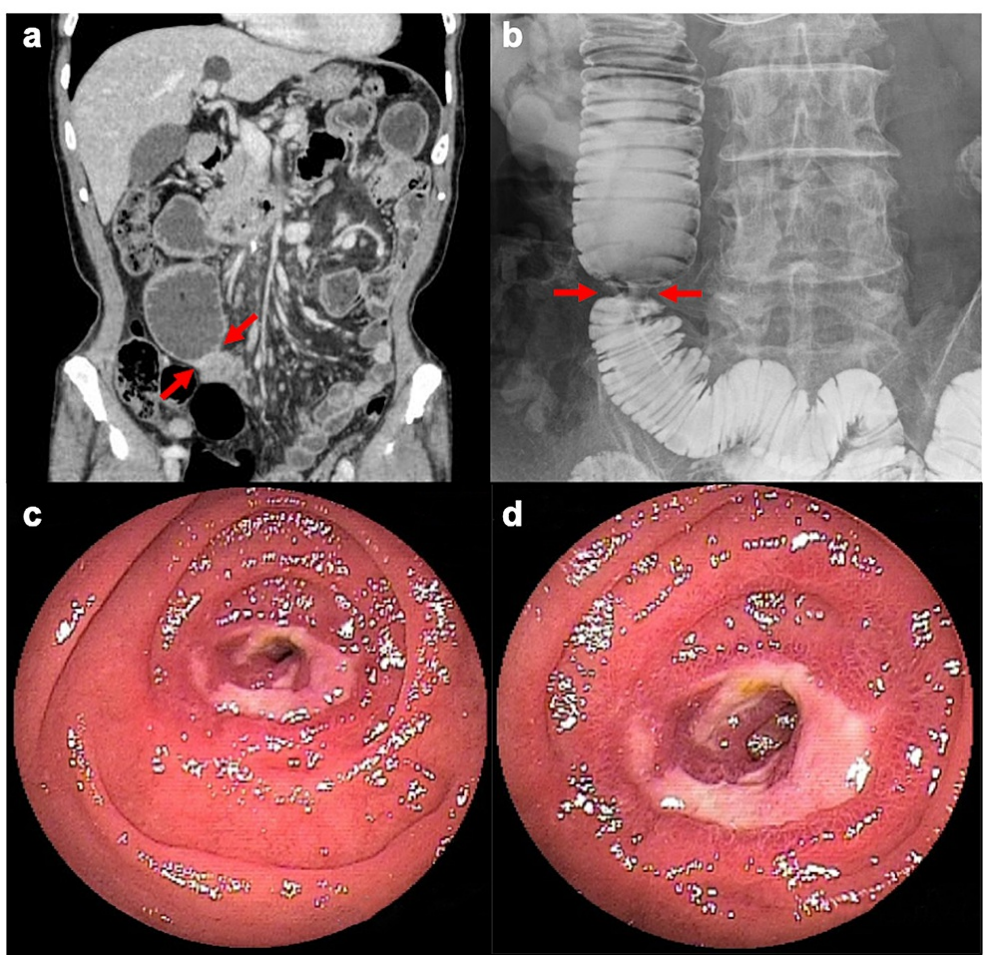
CT abdomen revealed a thickening of the small bowel wall with dilation of the proximal bowel (a, arrows). Small bowel series revealed severe jejunal stenosis (b, arrows). Antegrade double-balloon enteroscopy revealed severe stenosis with a circumferential ulcer in the jejunum (c, distant image; d, closeup image).

A standing plain abdominal radiograph revealed multiple air-fluid levels, which was suggestive of small bowel obstruction. Computed tomography (CT) of the abdomen revealed thickening of the small bowel wall with dilation of the proximal bowel (Figure 1a). Small bowel obstruction was improved by decompression using an ileus tube. A subsequent small bowel series revealed severe jejunal stenosis (Figure 1b). An antegrade double-balloon enteroscopy (DBE) also revealed severe stenosis with a circumferential ulcer in the jejunum (Figure 1c-1d). 

**Figure 2:**
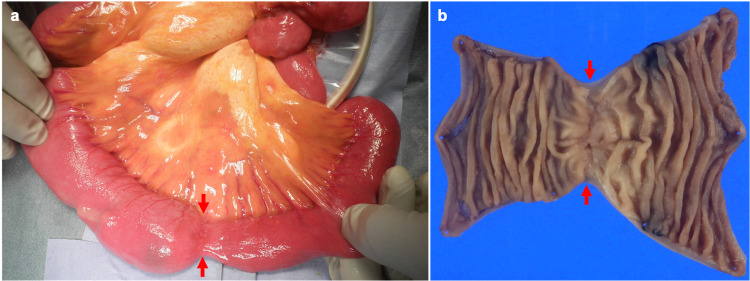
Intraoperative findings revealed a jejunal stenosis (a, arrows). Resected specimen revealed a circumferential ulcer at the location of the stenosis (b, arrows).

Although other differential diagnoses, including bowel tuberculosis, bowel ischemia, and idiopathic bowel stenosis, were considered, pathological examination of the biopsy specimens revealed non-specific findings. Esophagogastroduodenoscopy, colonoscopy, and retrograde DBE did not reveal any other lesions. After discussing with the patient, the stenosed portion of the small bowel was surgically resected. The intraoperative findings revealed jejunal stenosis (Figure 2a), and the resected specimen revealed a circumferential ulcer at the location of the stenosis (Figure 2b).

**Figure 3:**
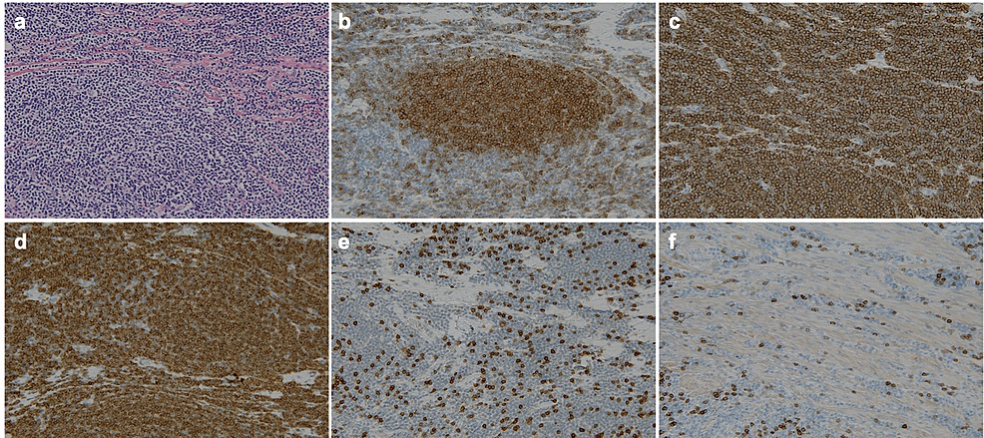
Pathological findings of the resected specimen revealed a concentration of small to medium-sized atypical lymphocytes (a, hematoxylin and eosin staining, ×200). The immunohistochemical staining was positive for CD10 (b), CD20 (c), and Bcl-2 (d) and negative for CD3 (e) and CD5 (f) (×200).

The pathological examination of the resected specimen revealed concentrated small to medium-sized atypical lymphocytes (Figure 3a) that were positive for CD10, CD20, and Bcl-2 and negative for CD3, CD5, and cyclin D1 in an immunohistochemical analysis (Figure 3b-3f); these results were suggestive of FL. Bone marrow biopsy was normal. Based on these findings, the patient was diagnosed with primary small bowel FL with clinical stage II_1_, according to the Lugano staging system for gastrointestinal lymphomas [6]. 

 He received no additional treatment postoperatively because fluorodeoxyglucose-positron emission tomography (FDG-PET) did not reveal lymph node swelling with significant FDG accumulation. He has had no recurrence for two years.

## Discussion

Gastrointestinal FLs were initially defined as a type of disease predominantly affecting the duodenum [7]. The representative morphology of duodenal lesions is multiple small, whitish, granular, or polypoid lesions [4]. Detecting the duodenal lesions using esophagogastroduodenoscopy had been the key diagnostic method in most patients with gastrointestinal FL. However, recent studies have revealed the frequent involvement of the jejunum and ileum in patients with gastrointestinal FL due to the development of novel endoscopic modalities to investigate small bowels, such as DBE or video capsule endoscopy (VCE). Among patients with gastrointestinal FL, the prevalence of cases with FL lesions in the jejunum or ileum reportedly ranges from 66.7-100% [8-13], and most of these lesions are located in the jejunum (75%) [5]. 

In the previous study that included 89 FL cases with jejunoileal involvement, small whitish nodules and polyp-like lesions were detected in 64 cases (71.9%) and 24 cases (27.0 %), respectively, while another morphology was detected in only one case (1.1%) [4]. Therefore, a circumferential ulcer with bowel stenosis, as in the present case, is an extremely rare morphology. 

To date, seven cases of gastrointestinal FL presenting with small bowel stenosis have been reported in literature published in English, including our case (Table 1) [14-19]. It was relatively dominant in females, and there was no difference in tumor location. In these cases, various strategies were used for managing the bowel stenosis caused by FL. Of these cases, five were treated with chemotherapy, and two with surgical resection alone. The chances of the initial response to chemotherapy are reportedly relatively high in patients with gastrointestinal FLs; therefore, chemotherapy is usually the first choice of treatment for primary gastrointestinal NHLs. However, the gastrointestinal FLs have a more indolent course compared to other sub-types of gastrointestinal NHL. Moreover, surgical resection allows the removal of the obstruction and helps reach a definite pathological diagnosis. Therefore, a wait and watch strategy was opted for after surgical resection in the two cases [20]. DBE-assisted endoscopic balloon dilatation was performed before chemotherapy in one case. Given the high response rate of gastrointestinal FLs to chemotherapy, endoscopic balloon dilatation for small bowel stenosis due to FL can be reasoned as management instead of surgical resection, as long as it can be performed safely [17].

**Table 2:** Case series of follicular lymphoma that presented with small bowel stenosis in the literature (English). *Lugano staging system for gastrointestinal lymphomas

No.	Reference no.	Author (year)	Age, years	Sex	Location	Diagnosis	Clinical stage*	Treatment
1	[14]	Yamada, et al. (2016)	72	Female	Ileum	Biopsy	II_2_	Chemotherapy
2	[15]	Kawasaki, et al. (2016)	63	Female	Jejunum	Biopsy	II_2_	Chemotherapy
3	[16]	Kawasaki, et al. (2020)	77	Female	Ileum	Biopsy	Not described	Surgery and chemotherapy
4	[17]	Magome, et al. (2020)	60	Male	Jejunum	Biopsy	II_1_	Endoscopic balloon dilatation and Chemotherapy
5	[18]	Osaki, et al. (2021)	73	Female	Ileum	Surgery	Not described	Surgery
6	[19]	Goto, et al. (2021)	79	Female	Jejunum	Biopsy	II_2_	Surgery and chemotherapy
7	-	Our case	73	Male	Jejunum	Surgery	II_1_	Surgery

## Conclusions

We reported a rare case of FL of the small bowel that presented with bowel stenosis. This case was not diagnosed from endoscopic findings and biopsies; thus, the lesion was diagnosed during surgical resection, resulting in a definitive diagnosis. The differential diagnosis of a circumferential ulcer with small bowel stenosis typically includes various diseases, such as bowel tuberculosis, NSAID-related ulcers, inflammatory bowel disease (e.g., Crohn’s disease), bowel ischemia, trauma, and idiopathic bowel stenosis. However, after ruling out all the likely diseases, atypical gastrointestinal FL lesions should be considered.
